# Differentiation-Dependent Secretion of Proangiogenic Factors by Mesenchymal Stem Cells

**DOI:** 10.1371/journal.pone.0035579

**Published:** 2012-04-20

**Authors:** Allison I. Hoch, Bernard Y. Binder, Damian C. Genetos, J. Kent Leach

**Affiliations:** 1 Department of Biomedical Engineering, University of California Davis, Davis, California, United States of America; 2 Department of Anatomy, Physiology, and Cell Biology, School of Veterinary Medicine, University of California Davis, Davis, California, United States of America; University of Udinem, Italy

## Abstract

Mesenchymal stem cells (MSCs) are a promising cell population for cell-based bone repair due to their proliferative potential, ability to differentiate into bone-forming osteoblasts, and their secretion of potent trophic factors that stimulate angiogenesis and neovascularization. To promote bone healing, autogenous or allogeneic MSCs are transplanted into bone defects after differentiation to varying degrees down the osteogenic lineage. However, the contribution of the stage of osteogenic differentiation upon angiogenic factor secretion is unclear. We hypothesized that the proangiogenic potential of MSCs was dependent upon their stage of osteogenic differentiation. After 7 days of culture, we observed the greatest osteogenic differentiation of MSCs when cells were cultured with dexamethasone (OM+). Conversely, VEGF protein secretion and upregulation of angiogenic genes were greatest in MSCs cultured in growth media (GM). Using conditioned media from MSCs in each culture condition, GM-conditioned media maximized proliferation and enhanced chemotactic migration and tubule formation of endothelial colony forming cells (ECFCs). The addition of a neutralizing VEGF_165/121_ antibody to conditioned media attenuated ECFC proliferation and chemotactic migration. ECFCs seeded on microcarrier beads and co-cultured with MSCs previously cultured in GM in a fibrin gel exhibited superior sprouting compared to MSCs previously cultured in OM+. These results confirm that MSCs induced farther down the osteogenic lineage possess reduced proangiogenic potential, thereby providing important findings for consideration when using MSCs for bone repair.

## Introduction

Cell survival requires appropriate access of nutrients and close proximity to capillaries, and thus, limited vascularization at an injury site remains a primary obstacle in tissue engineered therapies [1]. Transport limitations of tissue engineered constructs can be potentially resolved by establishing a new vasculature within the new tissue, and current studies aim to promote vascularization within growing tissues through vasculogenesis or angiogenesis. For example, smooth muscle cells have been transduced to overexpress vascular endothelial growth factor (VEGF), a potent mitogen which stimulates angiogenesis through proliferation and migration of endothelial cells [2].

Recent efforts have focused on coupling angiogenesis to bone repair since natural development and formation of the skeleton occurs in close proximity to vascular ingrowth [3]–[6]. Vasculature provides growth factors, hormones, cytokines, and chemokines required by the bone tissue, and vasculature disruption can lead to skeletal pathologies such as osteonecrosis and osteomyelitis [7]. Indeed, Huang *et al.* demonstrated that combining angiogenic and osteogenic factors exhibited increased bone formation *in vivo* compared to either factor alone [5], demonstrating the crucial interdependence of the two processes for bone repair.

Mesenchymal stem cells (MSCs) are an attractive cell source for use in tissue engineering due to their proliferative potential, multipotency, and immunomodulatory effects [8]–[10]. In addition to other phenotypes, MSCs can be induced toward the osteoblastic lineage when cultured in osteoinductive conditions [11] or placed on osteoconductive materials [12]. Furthermore, MSCs secrete multiple trophic factors that suppress the immune system [13], inhibit apoptosis [14], stimulate mitosis [15] and differentiation [8]–[10], and enhance angiogenesis [14], [16]. The benefits of MSC trophic factor secretion have been confirmed using co-cultures with endothelial cell populations [3], as well as conditioned media [4] from MSCs. When transplanted *in vivo*, MSCs enhance and accelerate angiogenesis and stabilize new blood vessels [16].

The composition of cell culture media has a significant effect on MSC cytokine expression [17], [18]. Media supplemented with cocktails of specific components at precise concentrations can effectively drive MSCs toward distinct phenotypes such as bone, cartilage, and fat [11]. To stimulate the production of inorganic and organic components in bone, MSCs can be cultured in media supplemented with combinations of ascorbic acid, β-glycerophosphate, and dexamethasone. Specifically, ascorbic acid increases synthesis of collagen and extracellular matrix and β-glycerophosphate provides phosphate ions to promote hydroxyapatite formation [19]. Dexamethasone, a potent glucocorticoid, also facilitates mineralization by increasing alkaline phosphatase activity and cyclic adenosine monophosphate (cAMP) responses to parathyroid hormone (PTH) and prostaglandin [20], [21]. However, not all differentiation protocols include dexamethasone in the osteoinductive cocktail [22], [23], since glucocorticoids have been shown to inhibit collagen type I and osteocalcin expression in osteoblasts [24]. The inclusion of cocktail components directs osteogenic differentiation of MSCs to varying degrees, but the contribution of osteogenic differentiation on trophic factor secretion is poorly understood [17].

We hypothesized the secretion of proangiogenic trophic factors by MSCs was a function of the stage of differentiation down the osteogenic lineage. To explore this hypothesis, we measured the quantity and bioactivity of endogenous proangiogenic factors from MSCs when osteogenically induced in different culture media. Furthermore, we examined the capacity of osteogenically-induced MSCs to contribute to capillary sprouting and stabilization when co-cultured with endothelial cells in fibrin gels. The results of these studies provide an improved understanding of the balance between MSC differentiation and trophic factor secretion for use as a cell-based therapy for bone regeneration and repair.

## Results

### Dexamethasone enhances osteogenic differentiation

**Figure 1:**
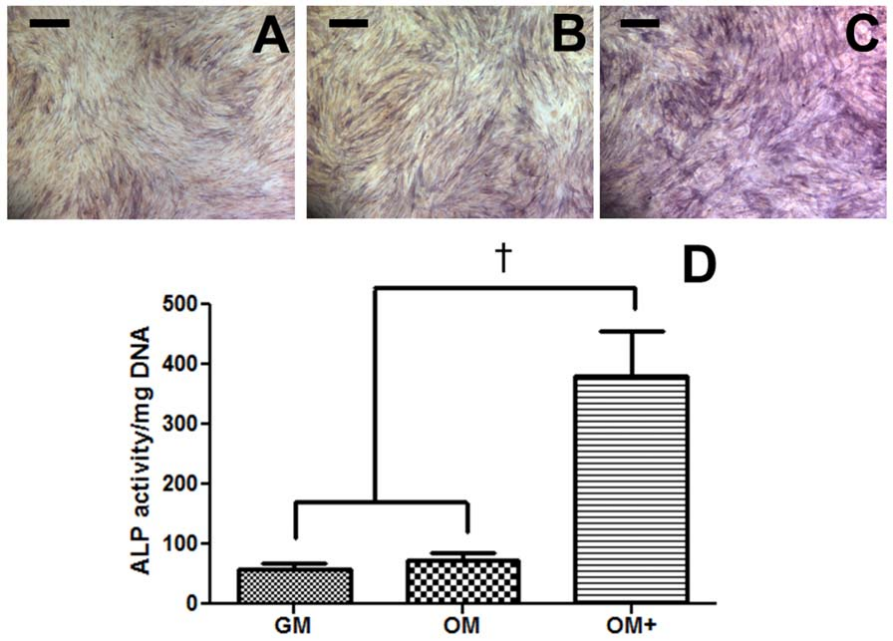
Cytochemical stains and biochemical quantification of ALP activity in MSCs after 7 days. MSCs cultured in (A) GM, (B) OM, and (C) OM+. Scale bars represent 200 µm. (D) Quantification of intracellular ALP activity in MSCs as a function of media composition. Chart values represent mean ± SD for n = 4; †*p*<0.001 vs. GM and OM.

MSCs were cultured for 7 days in growth media (GM), osteogenic media (OM), or osteogenic media supplemented with dexamethasone (OM+) (**Table 1**) and analyzed for alkaline phosphatase (ALP) activity, an early marker of osteogenic differentiation. The addition of dexamethasone to the culture media increased intracellular ALP production. Compared to MSCs in GM (**Fig. 1A**) or OM (**Fig. 1B**), staining intensity for ALP in MSC was increased for cells in OM+ (**Fig. 1C**). MSCs cultured in OM did not exhibit significant increases in ALP activity after 7 days of culture. These qualitative observations were corroborated by quantification of intracellular ALP activity (**Fig. 1D**).

**Table 1:** Cell culture media and composition.

Name	Media	Supplements
GM	α-MEM, 10% FBS, 1% P/S	-
OM	α-MEM, 10% FBS, 1% P/S	10 mM βGP, 50 µg/mL A2P
OM+	α-MEM, 10% FBS, 1% P/S	10 mM βGP, 50 µg/mL A2P, 10 nM dexamethasone
EBM2	EBM2	-
GF-Def EGM2	EBM2, 10% FBS, 1% P/S	Hydrocortisone, gentamycin-1000, EGF, heparin
EGM2	EBM2, 10% FBS, 1% P/S	Hydrocortisone, gentamycin-1000, EGF, heparin, VEGF, FGF, IGF

### Proangiogenic potential of MSCs is impaired by osteogenic differentiation

**Figure 2:**
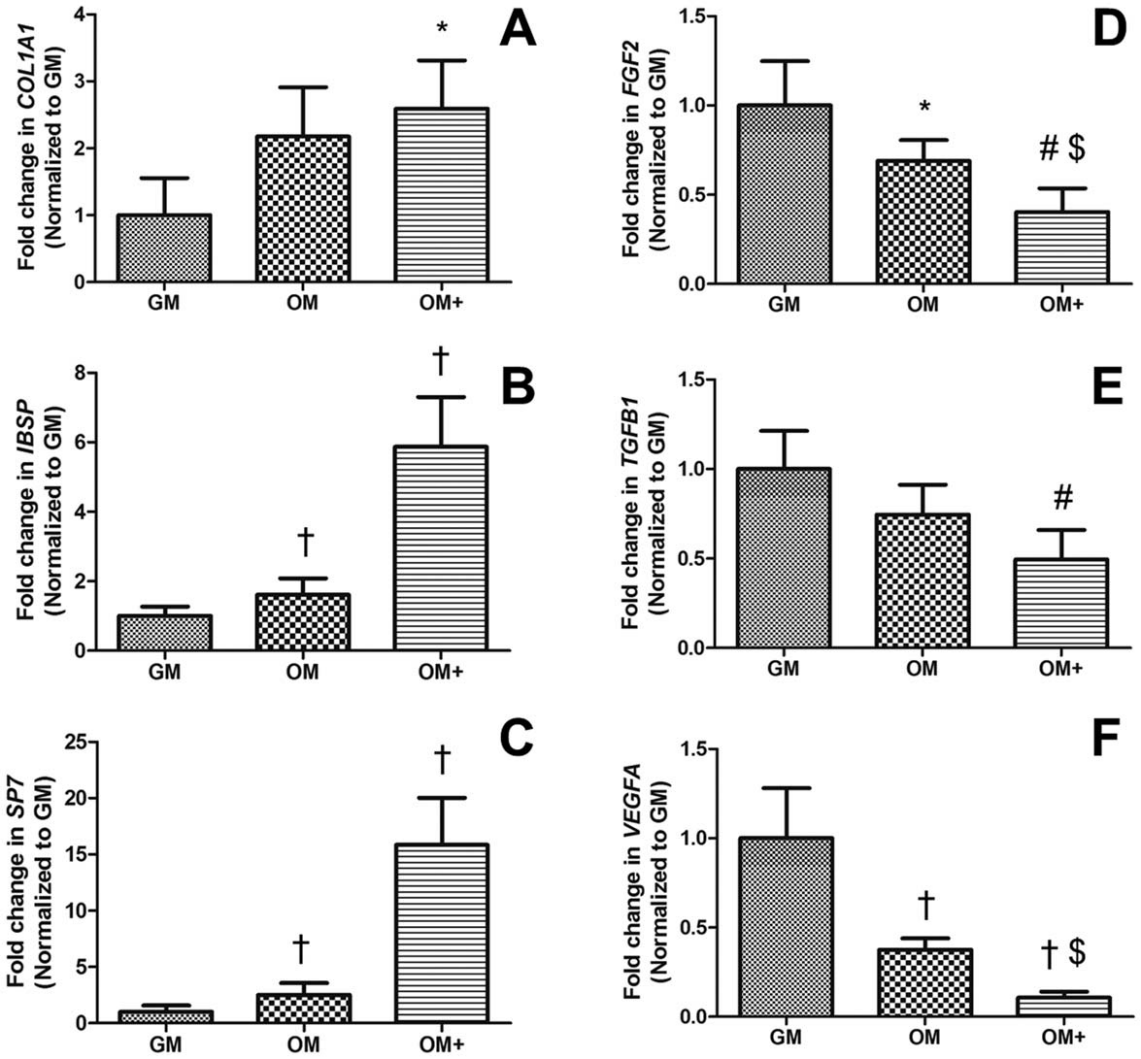
qPCR expression of osteogenic and angiogenic marker genes in MSCs after 7 days. Fold increase in (A) *COL1A1*, (B) *IBSP*, (C) *SP7*, (D) *FGF2*, (E) *TGFB1*, and (F) *VEGFA*. Data were normalized to *RPL13* transcript level and then normalized to GM. Chart values represent mean ± SD for n = 4; †*p*<0.001 vs. GM; #*p*<0.01 vs. GM; *p<0.05 vs. GM; $*p*<0.05 vs. OM.

After 7 days, MSCs cultured in each media composition were analyzed for expression of angiogenic (*FGF2*, *PDGFB*, *TGFB1*, and *VEGFA*) and osteogenic markers (*COL1A1*, *IBSP*, and *SP7*). Cells cultured in OM+ exhibited increased expression of osteogenic genes (**Fig. 2A–C**) and conversely, decreased expression of angiogenic genes (**Fig. 2D–F**). In all groups, expression of *PDGFB* was not detected (*data not shown*).

**Figure 3:**
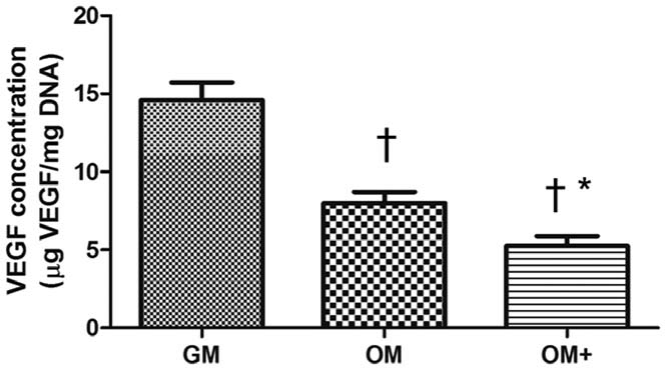
VEGF secretion by MSCs after 7 days of differentiation. Chart values represent mean ± SD for n = 4; †*p*<0.001 vs. GM; *p<0.05 vs. OM.

**Figure 4:**
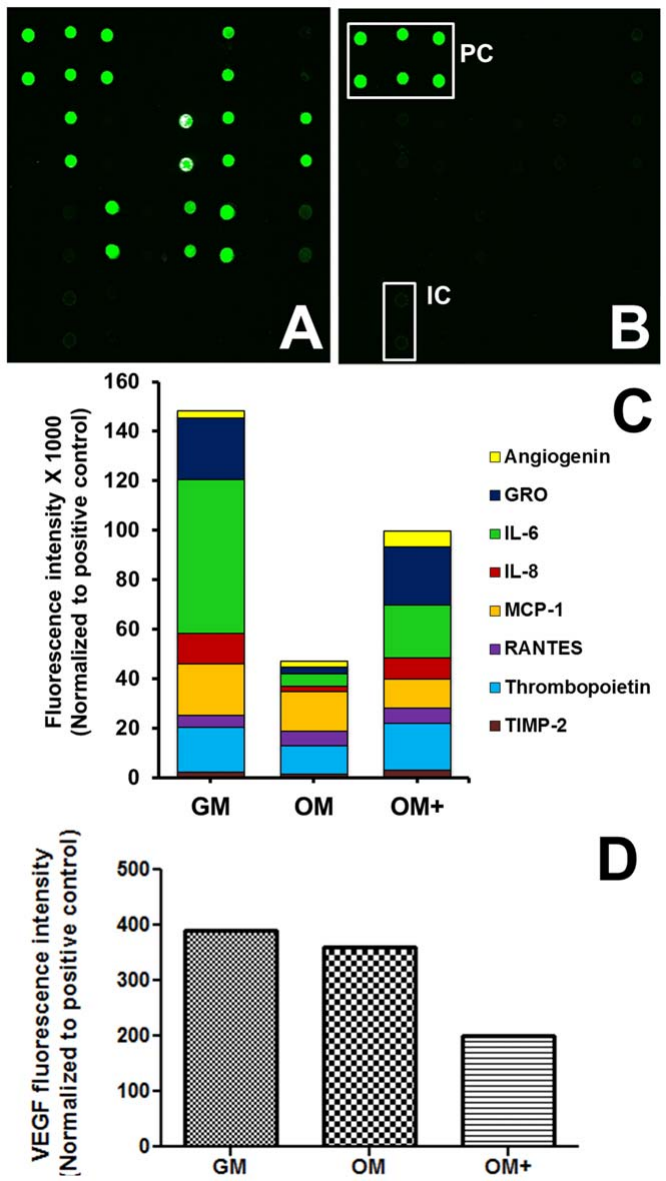
Quantification of angiogenic cytokines in conditioned media. (A) Conditioned GM and (B) original unconditioned GM demonstrate serum contains trace amounts of angiogenic cytokines save the positive (PC) and internal controls (IC). (C) Stacked column chart of fluorescence (×1000) of predominant angiogenic cytokines and (D) VEGF fluorescence as a function of conditioned media. Data is presented as median subtracted background fluorescence intensity and normalized to positive controls and excluding inherent serum levels for pooled conditioned media for n = 4.

The concentration of secreted VEGF was greatest for MSCs cultured in GM. The addition of osteogenic supplements to the culture media decreased VEGF secretion (**Fig. 3**). Cells cultured in OM+ secreted VEGF quantities that were significantly less than MSCs cultured in GM or OM. We quantified the production of proangiogenic proteins in response to GM, OM, or OM+. Serum alone did not exhibit detectably distinct levels of angiogenic cytokines compared to conditioned media (**Fig. 4A, B**). A stacked column chart of the predominant angiogenic cytokines (100–1000-fold greatest) reveals MSCs conditioned in GM secreted the highest concentrations of angiogenic cytokines, whereas MSCs conditioned in OM secreted the lowest (**Fig. 4C**). Concentrations of most angiogenic factors decreased as media included more osteogenic supplements. In contrast, angiogenin and RANTES (CCL5) concentration notably increased. VEGF concentration determined through the angiogenic protein array decreased with osteogenic media (**Fig. 4D**).

### ECFC proliferation, migration, and tubule formation is altered by MSC conditioned media

**Figure 5:**
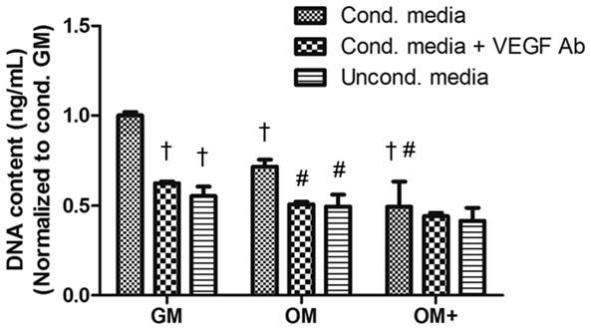
Endothelial cell proliferation measured through DNA content in response to MSC-conditioned media. Data are presented as fold decrease from conditioned GM. Neutralizing VEGF_165/121_ antibody added at 10 µg/mL to conditioned media. Chart values represent mean ± SD for n = 3–4; †*p*<0.001 vs. conditioned GM; #*p*<0.01 vs. conditioned OM.

ECFCs exhibited the greatest proliferation when stimulated with conditioned media from cells in GM, followed by OM and OM+ (**Fig. 5**). The addition of a neutralizing VEGF_165/121_ antibody significantly attenuated proliferation in GM and OM, whereas the effect was less pronounced for OM+. Conditioned GM and OM induced significantly greater ECFC proliferation than unconditioned GM and OM, respectively, indicating that secretion of trophic factors from MSCs, and not media composition alone, was responsible for the observed changes in ECFC proliferation. No significant differences in proliferation were observed among unconditioned medias.

**Figure 6:**
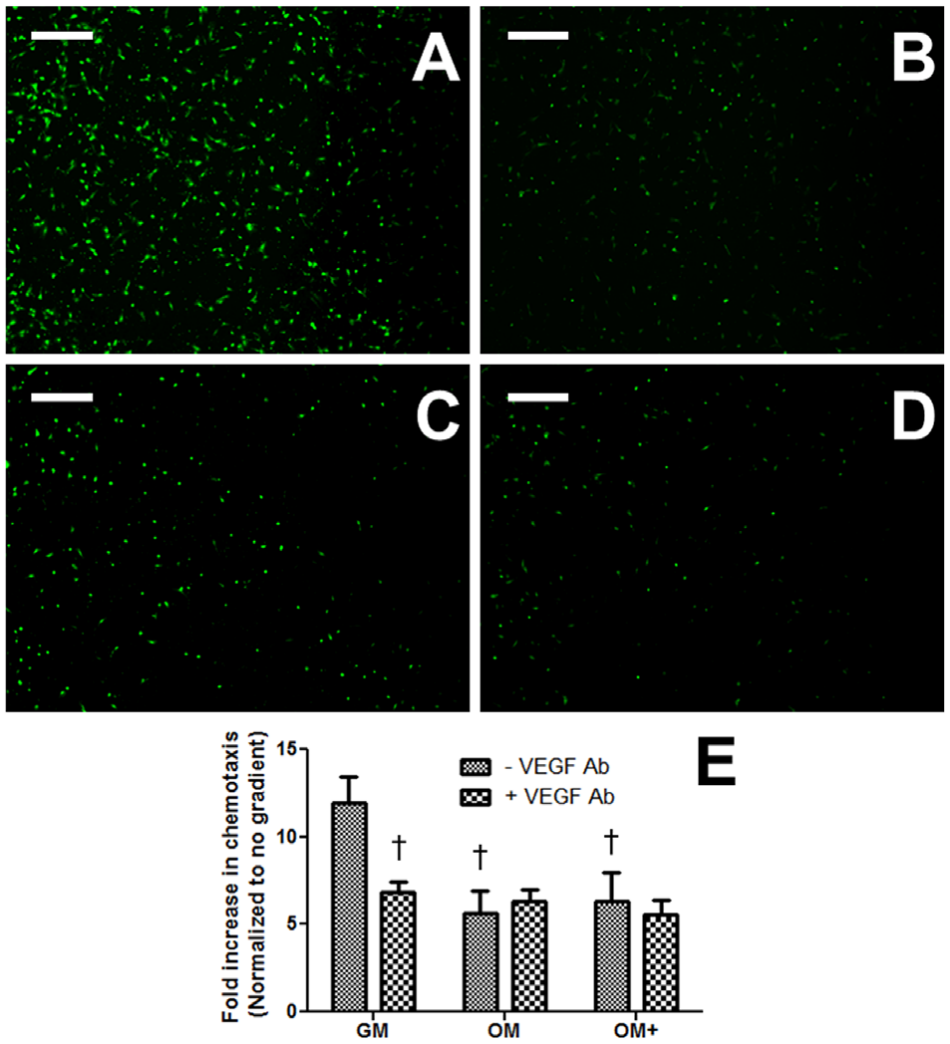
Endothelial cell chemotaxis due to MSC-conditioned media. (A) GM, (B) GM with neutralizing 10 µg/mL VEGF_165/121_ antibody, (C) OM, or (D) OM+. Images are representative of 4 wells. Scale bars represent 200 µm. (E) Fold increase in chemotaxis normalized to no gradient. Chart values represent mean ± SD for n = 4; †*p*<0.001 vs. GM.

ECFC migration toward conditioned GM through a transwell insert increased 12-fold compared to no gradient (**Fig. 6A, E**), and chemotaxis to GM was significantly greater than ECFC migration toward conditioned OM (**Fig. 6C**) and OM+ (**Fig. 6D**). The addition of a neutralizing VEGF_165/121_ antibody significant reduced ECFC migration in response to conditioned GM (**Fig. 6B**).

**Figure 7:**
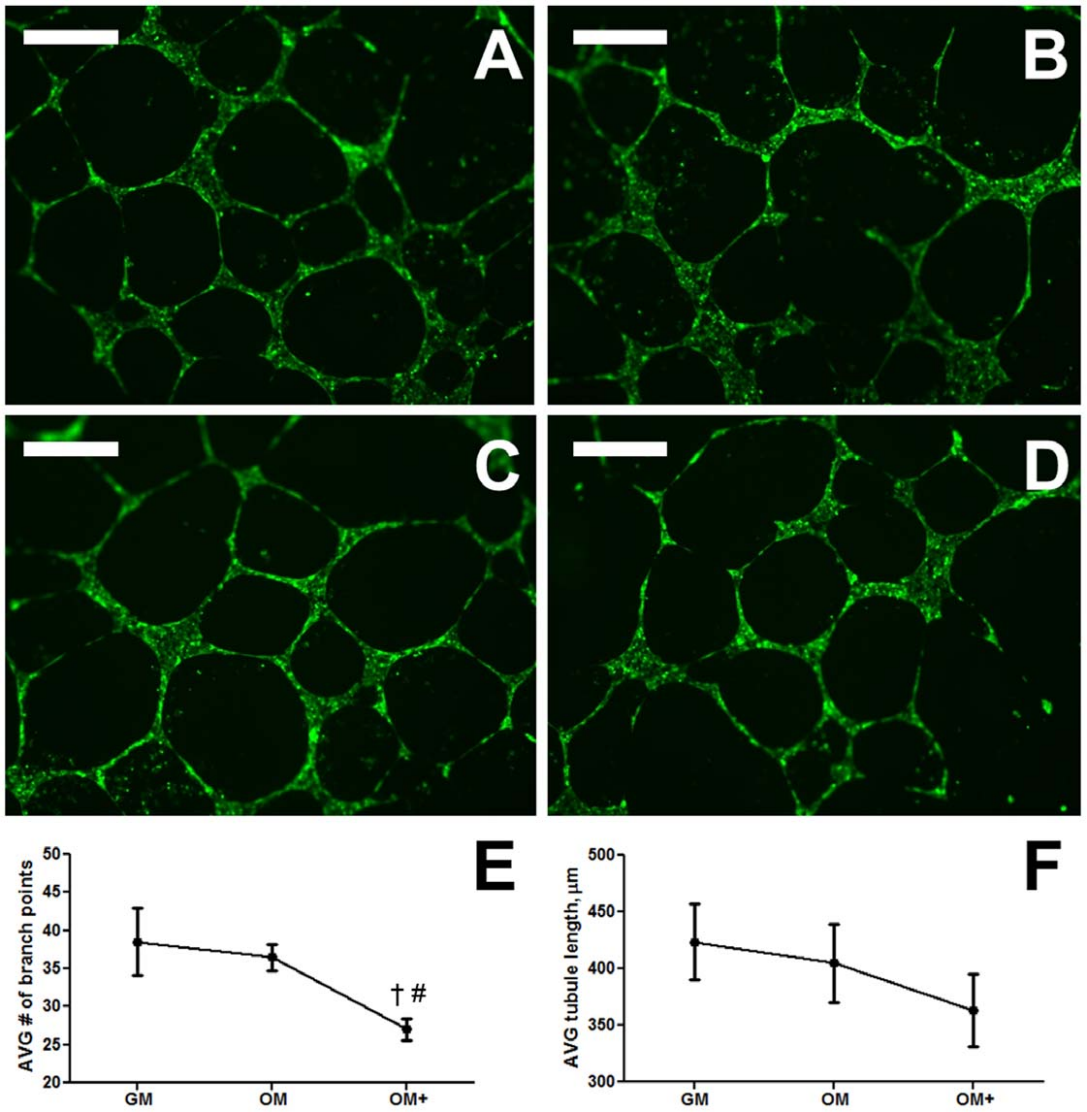
Endothelial cell tubulogenesis on Matrigel in the presence of MSC-conditioned media. (A) GM, (B) GM with neutralizing 10 µg/mL VEGF_165/121_ antibody, (C) OM, or (D) OM+. Images are representative of 4 wells. Scale bars represent 500 µm. (E) Average number of branch points and (F) Average tubule length in field of view. Chart values represent mean ± SD for n = 4; †*p*<0.001 vs. GM; #*p*<0.01 vs. OM.

Using Matrigel as a basement membrane, we observed similar branch point frequency in ECFC-derived tubules with conditioned media from MSCs cultured in GM (**Fig. 7A**) or OM (**Fig. 7C**). We quantified fewer branch points in ECFCs grown in media from OM+-cultured MSCs (**Fig. 7D**). Quantification of branch points as an indicator of sprouting revealed a significant reduction for ECFCs cultured in OM+-conditioned media (**Fig. 7E**), and we observed a similar trend when quantifying average tubule length (**Fig. 7F**). The addition of neutralizing VEGF_165/121_ antibody did not significantly alter any tubule parameters except the arbitrary number of disrupted tubules, which is qualitatively illustrated in the comparison between **Fig. 7A** and **Fig. 7B**.

### Osteogenic differentiation reduces capillary sprouting

**Figure 8:**
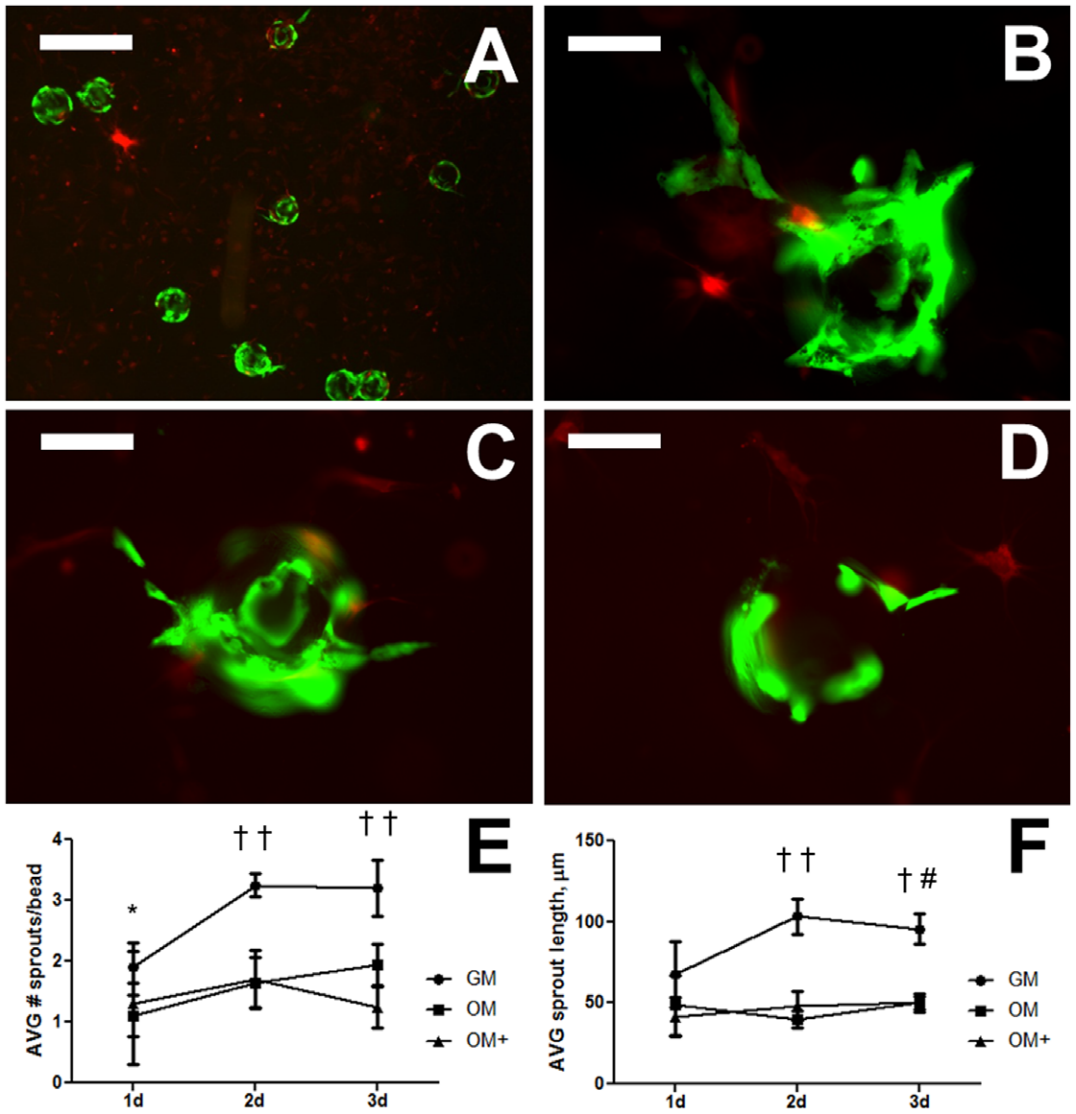
Endothelial cell sprouting from microcarrier beads in co-culture with conditioned MSCs. (A) MSCs co-cultured with GFP-ECFCs seeded on microcarrier beads are uniformly distributed within fibrin gels after 1 day. Representative sprouting observed when cultured with MSCs conditioned in (B) GM, (C) OM, and (D) OM+ after 2 days. Images are representative of 4 wells. Scale bar in A represents 500 µm; scale bars in B–D represent 100 µm. (E) Average number of sprouts per bead; (F) Average sprout length. Chart values represent mean ± SD for n = 4; †*p*<0.001 vs. OM and/or OM+; #*p*<0.01 vs. OM, *p<0.05 vs. OM+.

GFP-ECFCs seeded on microcarrier beads were co-cultured with CellTracker™ Orange-labeled MSCs, pre-conditioned in each media for 7 days, and uniformly distributed throughout the fibrin gel (**Fig. 8A**). Co-cultures with GM-conditioned MSCs (**Fig. 8B**) exhibited visually improved sprouting compared to MSCs cultured in OM (**Fig. 8C**) and OM+ (**Fig. 8D**). Quantitative analysis revealed GM-conditioned MSCs increased average sprout number per bead (**Fig. 8E**) and average sprout length (**Fig. 8F**) compared to other cells. For all groups, sprouting parameters increased until 2 days and plateaued by 3 days.

## Discussion

Human MSCs were differentiated to varying degrees in osteogenic media to test our hypothesis that the secretion of proangiogenic factors by MSCs was a function of their stage of osteogenic differentiation. When stimulating ECFCs to measure proangiogenic potential, conditioned media from MSCs cultured in growth media resulted in increased angiogenic stimulation compared to MSCs in either osteogenic media. Moreover, MSCs differentiated in dexamethasone-containing osteogenic media (OM+) exhibited minimal angiogenic potential for all studies. These findings indicate that osteogenically-differentiated MSCs have a limited capacity to support angiogenesis, and thus, these data highlight the importance of considering modulations of MSC trophic factor secretion due to culture conditions.

The addition of dexamethasone to osteogenic supplements significantly increased differentiation of MSCs within 7 days compared to growth media or osteogenic media without dexamethasone. ALP activity, along with *COL1A1*, *IBSP*, and *SP7* gene expression, was greatest in cells maintained in OM+. MSCs in osteogenic media lacking dexamethasone did not exhibit significant increases in osteogenic markers after 7 days, suggesting that longer periods of culture are required to effectively differentiate MSCs without this corticosteroid. Dexamethasone stimulates MSC differentiation into osteoblasts by increasing ALP activity among other mechanisms [20], [21]. However, not all protocols utilize this molecule as an osteogenic supplement [22], [23] because it decreases MSC production cytokines such as leukemia inhibitory factor (LIF), interleukin-6 (IL-6), and interleukin-11 (IL-11), which decrease osteocalcin and may inhibit osteoblast differentiation [17].

Angiogenesis, a crucial event in bone formation, involves capillary-forming endothelial cells that are stimulated by proangiogenic factors [25]. MSCs secrete numerous trophic and angiogenic factors such as VEGF, a potent soluble cue that induces migration and proliferation of endothelial cells and stimulates neovascularization [26]. In these studies, MSCs cultured in GM secreted the highest concentrations of VEGF. In contrast, MSCs cultured in osteogenic media downregulate gene expression of the proangiogenic factors *VEGFA*, *TGFB1*, and *FGF2* [27]. ECFCs exhibited the greatest mitogenic response when stimulated with conditioned media of MSCs cultured in GM, which was shown to contain the highest concentration of VEGF and other proangiogenic cytokines. Interleukins 6 and 8 (IL-6 and IL-8) are prominent angiogenic cytokines found in conditioned growth media are generally secreted by leukocytes, macrophages, and other cell types such as MSCs as part of the immune response to regulate hematopoietic cell development and differentiation. Both IL-6 and IL-8 concentrations decreased from GM to OM+, which confirms evidence that dexamethasone decreases IL-6 production [17]. Interaction among these and other angiogenic cytokines and the subsequent autocrine and paracrine effects merits further exploration.

OM+-cultured MSCs were most differentiated toward osteoblasts and secreted minimal VEGF. VEGF-A is secreted in a differentiation-dependent manner during osteogenesis and peaks upon mineralization after osteogenic culture for 7 weeks [28] and 18 days [29]; however, the effects of VEGF secretion in direct application to endothelial cells have not been thoroughly investigated. VEGF-A exists in 5 different isoforms. Whereas VEGF_165_ is detected at all stages during early osteogenesis, VEGF_121_ principally serves as the primary angiogenic factor in early osteogenesis [30]. A polyclonal VEGF_165/121_ neutralizing antibody was added to the proliferation, migration, and Matrigel tubule formation assays. The antibody significantly inhibited ECFC proliferation and migration in response to conditioned GM, demonstrating that VEGF secreted by MSCs in conditioned media is responsible for its subsequent angiogenic effects on endothelial cells. While the antibody disrupted tubule formation, it did not lead to a significant decrease in number of tubule branches or length of tubules. Such data is in agreement with previous reports in which the addition of VEGF neutralizing antibodies did not affect average number of tubule branches [31]. The ability of VEGF to promote vascularization has spawned therapies to incorporate the protein either through entrapment in scaffolds [32], [33] or bolus injection [34]; however, both methods expose severe limitations. The short half-life of VEGF leads to rapid biodegradation and a bolus injection may cause hypertension [2]. Therefore, sustained, localized delivery of physiological concentrations of VEGF through MSC secretion offers a promising alternative to stimulate angiogenesis.

Bischoff *et al.* demonstrated that dexamethone-containing osteogenic media (OM+) stimulated secretion of the CXC family of angiogenic chemokines, which are classified by a Glu-Leu-Arg (ELR) motif located at the N-terminus [35]. The greatest endothelial cell tubule branching was observed with OM+ conditioned media, which is in disagreement with the results of this study. The discrepancy may be due to differences in plating density, culture duration, and dilution of conditioned media. Schubert *et al.* reported enhanced VEGF secretion from porcine bone marrow-derived MSCs (BM-MSCs) following osteogenic differentiation for 26 days compared to undifferentiated porcine BM-MSCs [36]. While the effect of long-term differentiation on the secretion of trophic factors is an important consideration, it is necessary to minimize the duration of *ex vivo* culture and expansion to capitalize on the acute application of cell-based therapies for tissue engineering, thus motivating our examination of trophic factor secretion over a shorter 7 day period.

Co-cultures of MSCs and endothelial cells enhance angiogenesis in bone repair where establishment of an adequate vasculature remains a significant challenge [37], [38]. In these studies, angiogenesis was also assessed using differentiated MSCs and ECFCs in co-culture. Similar to our findings for tubule formation, GM-conditioned MSCs were responsible for improving angiogenic parameters, even in the absence of exogenous mitogenic growth factors. Cell-cell contact was likely established in this sprouting study because MSCs were seeded at high density within the gel and are detected in close proximity to endothelial cells. Importantly, these results validate the findings that co-cultures of MSCs and endothelial cells can induce angiogenesis without the addition of exogenous growth factors [37].

Our results provide detailed insight into the angiogenic trophic factor secretion of MSCs as a function of early differentiation toward osteoblasts. Moreover, these studies decouple the proangiogenic potential of MSCs from stimulation with osteogenic supplements. Future work entails examining the proangiogenic potential of MSCs differentiated for longer durations when co-cultured with endothelial cells. Additionally, the precise role of cell-cell signaling implicated in the angiogenic process must be investigated.

## Materials and Methods

### Cell culture

Human bone marrow-derived MSCs (Lonza) were expanded without further characterization in alpha-modified minimum essential media (α-MEM, Invitrogen) supplemented with 10% fetal bovine serum (FBS, JR Scientific) and 1% penicillin and streptomycin (P/S, Mediatech). Cells were cultured under standard conditions (37°C, 5% CO_2_, 21% O_2_) until use at passage 6. MSCs were seeded at 30,000 cells/cm^2^ in 12-well plates and allowed to attach overnight. Thereafter, cells were cultured for 7 days in growth media (GM: α-MEM, 10% FBS, 1% P/S), osteogenic media (OM: GM supplemented with 10 mM β-glycerophosphate (βGP) and 50 µg/mL ascorbate-2-phosphate (A2P)), or osteogenic media supplemented with 100 nM dexamethasone (OM+) [39], all from Sigma. For each experiment, GM, OM, and OM+ aliquots were derived from the same batch of serum containing α-MEM to ensure serum consistency. Media changes were performed every 3 days.

Human umbilical cord blood endothelial colony forming cells (ECFCs) were isolated using a protocol approved by the Institutional Review Board of the Indiana University School of Medicine as previously described [40]. Adherent ECFCs were cultured under standard conditions in T-75 culture flasks coated with 5 µg/cm^2^ rat tail collagen I (BD Biosciences) in SingleQuot-supplemented endothelial cell growth media 2 (EGM2) (Lonza) containing 10% FBS and 1% P/S. Growth factor-deficient media (GF-Def EGM2) was prepared with serum-containing EGM2 but lacking VEGF, FGF, and IGF, as these growth factors had the most profound effect on mitogenicity (*data not shown*). Media composition and nomenclature is detailed in **Table 1**.

### Osteogenic potential

Intracellular alkaline phosphatase (ALP) expression was qualitatively observed after 7 days using a cytochemical stain and quantitatively assessed using a colorimetric assay as previously described [41]. For quantification, cells were rinsed in PBS, collected in 500 µL passive lysis buffer (Promega), and sonicated at 40% amplitude for 5 s. Changes in ALP activity were determined from the lysate using a routine *p*-nitrophenyl phosphate (PNPP) colorimetric assay wherein absorbance is measured at 405 nm. ALP activity was normalized to total DNA content from the same cell lysate as quantified using Quant-iT PicoGreen dsDNA Assay Kit (Invitrogen) according to the manufacturer's protocol. Gene expression as a function of osteogenic differentiation was assessed by quantitative PCR as described below.

### Quantitative PCR

Total RNA was collected using the RNeasy Mini kit (Qiagen) and 480 ng of total RNA was reverse-transcribed with the QuantiTect Reverse Transcription Kit (Qiagen). qPCR was performed using TaqMan1 Universal PCR Master Mix (Applied Biosystems) on a Mastercycler1 realplex2 (Eppendorf). Primers and probes consisted of *COL1A1* (Hs01076777_m1), *FGF2* (Hs00266645_m1), *IBSP* (Hs00173720_m1) *PDGFB* (Hs00234042_m1), *SP7* (Hs01866874_s1), *TGFB1* (Hs00998133_m1), and *VEGFA* (Hs00900055_m1) (Applied Biosystems). Amplification conditions were 50°C for 2 min, 95°C for 10 min, followed by 40 cycles at 95°C for 15 s and 60°C for 1 min. Quantitative PCR results were normalized to *RPL13* (Hs00204173_m1) transcript level to yield ΔCt. Fold changes between GM were calculated to yield ΔΔCt. Data is presented as 2^−ΔΔCt^ and standard deviation is calculated according to the manufacturer's protocol.

### Proangiogenic protein secretion

The concentration of VEGF in conditioned media of MSCs undergoing differentiation was determined using a human VEGF ELISA kit according to the manufacturer's protocol (R&D Systems). After 7 days of differentiation, fresh media was added and conditioned for 48 hrs prior to collection. Protein secretion was normalized to DNA content of cells within the well. Quantification of angiogenic proteins within conditioned media was performed using a Human Angiogenesis Antibody Array (RayBio). Pooled conditioned media from 4 replicates was applied to one array well. Protein quantification was performed with a GenePix 4000B scanner and associated GenePix Pro software (Molecular Devices) to calculate median minus background fluorescent intensity at 532 nm for each cytokine. Data is normalized to positive controls and excludes protein concentrations found in serum contained in unconditioned GM.

### ECFC proliferation in conditioned media

The proliferative response of ECFCs to proangiogenic stimuli was determined similar to previous studies [42]. Briefly, ECFCs were seeded at 7,500 cells/cm^2^ in EGM2 in 12-well collagen-coated culture plates and allowed to attach overnight. The next day, culture media was refreshed with a 1∶4 volume ratio of MSC-conditioned media to GF-Def EGM2 and cultured for 72 hrs. Each well was then rinsed with PBS to remove non-adherent cells and debris. Proliferation was analyzed using Quant-iT PicoGreen dsDNA Assay Kit (Invitrogen). Cells cultured in complete EGM2 served as the positive control, while cells cultured in EBM2 served as the negative control. Cells cultured in 1∶4 volume ratio of unconditioned GM, OM, or OM+ to GF-Def EGM2 also served as a control. For proliferation, migration, and tubule formation experiments, exogenous VEGF in the conditioned media was neutralized by the saturating addition of 10 µg/mL anti-human VEGF_16512_/antibody (AB-293-NA, R&D Systems) [31] diluted in PBS. Control groups were administered an equivalent volume of PBS.

### ECFC migration and tubule formation in conditioned media

The potential of MSC-conditioned media to stimulate ECFC migration [42] and tubule formation [43] was determined as previously described. For migration, 24-well FluoroBlock™ transwell inserts (3 µm pore size, BD Biosciences) were coated with a thin layer of gelatin solution (0.1% fish oil gelatin, Sigma). ECFCs (1×10^5^ cells/well) were seeded on the top of the transwell inserts in 300 µL 1∶4 unconditioned GM, OM, or OM+ to GF-Def EGM2. Transwell inserts were then placed over 1 mL 1∶4 conditioned GM, OM, or OM+ to GF-Def EGM2 to create a positive chemotactic gradient. The no-gradient control consisted of 1 mL GF-Def EGM2. Plates were then incubated for 24 hrs. Cells that migrated through the transwell insert were stained *via* calcein AM (3 µg/mL in PBS) for 30 min, and fluorescence was quantitated using a microplate reader (Synergy HTTR) at 485/530 nm and qualitatively captured using fluorescence microscopy. For tubule formation, 100 µL of Growth Factor Reduced Matrigel (BD Biosciences) was pipetted into 48-well plates and allowed to gel at 37°C for 1 hr. ECFCs transduced to express green fluorescent protein using a lentiviral vector (GFP-ECFCs) were seeded on Matrigel at 30,000 cells/cm^2^ in GF-Def EGM2, and a mixture of conditioned media and GF-Def EGM2 was added in a 1∶4 volume ratio. Cells were cultured for 16 hrs, and images of tubule formation were captured by fluorescence microscopy. We calculated average tubule length, average branch number, and average number of disrupted tubules from 25 tubules per field of view using NIH ImageJ in 4 independent experiments. Prior to analysis, images were randomized and descriptive markers were removed to ensure unbiased evaluation.

### ECFC sprouting in fibrin co-cultures with conditioned MSCs

The proangiogenic potential of osteogenically-induced MSCs when co-cultured with ECFCs was further examined using a sprouting assay as previously described [44]. Briefly, Cytodex® microcarrier beads (133–215 µm, Sigma) were swollen in PBS, sterilized in 70% ethanol overnight, and washed repeatedly with EGM2. In a flow cytometry sample tube, 2.5 mL of GF-Def EGM2, 5,000 beads, and 4×10^6^ GFP-ECFCs were combined and agitated every 20 min in an incubator. After 4 hrs, the contents were transferred to a T-25 flask containing 2.5 mL of fresh GF-Def EGM2 and incubated overnight in standard cell culture position. Conditioned MSCs were fluorescently tagged with CellTracker™ Orange (Invitrogen) according to the manufacturer's protocol. In GF-Def EGM2, 2 mg/mL fibrinogen (Calbiochem) was prepared. The fibrinogen solution (400 µL) was mixed with 50 µL of 50,000 MSCs resuspended in GM and 100 µL of the GFP-ECFC-bead suspension and subsequently polymerized with 0.625 U/mL thrombin (Sigma) in a single well of a 24-well plate. Gels were incubated for 25 min in standard culture conditions prior to addition of 1 mL GF-Def EGM2. Media was changed the following day and every other day thereafter. Images were captured using fluorescence microscopy for 3 days. Sprouting was quantified using previously described methods [45]. Briefly, 5 beads per gel in each of 4 gels were analyzed for each condition. Vessel segments for each bead were traced, counted, and quantified using ImageJ. The resulting values of average number of sprouts and average sprout length for 20 beads per condition were tabulated and averaged.

### Statistical analysis

Data are presented as mean ± standard deviation from at least three independent experiments. Statistical significance was assessed by one-way ANOVA followed by a Student-Newman–Keuls post hoc test, and *p*-values<0.05 were considered statistically significant. Statistical analysis was performed using GraphPad Prism®4 analysis software (GraphPad Software).
